# Supplementary material for: Precise language responses versus easy rating scales—Comparing respondents’ views with clinicians’ belief of the respondent’s views
Source: PLoS One. 2023 Feb 15;18(2):e0267995. doi: 10.1371/journal.pone.0267995 (PMC9931093; doi:10.1371/journal.pone.0267995)
Supplement: S1 Appendix — (DOCX) [file pone.0267995.s001.docx]

**Appendix**:

Instructions to respondents:

In this study we are interested in what you think about different responses formats when describing your level of depression. First you will be asked to answer four types of questions about depression with different response formats, including the following. The response formats are: fill out a *Rating* scale, *Select* 5 descriptive words from a list of 30 words, write 5 descriptive *Words*, and write a *Text* of 20 to 1000 words. Second, you will be asked a series of questions about how you perceived answering questions with the different response formats. Try to weigh the strength, and the number of aspects, so that it reflects your overall personal level of depression, or lack of depression.

Instruction to researchers:

In this study we are interested in what you think about different response formats when participants describe their level of depression. First, you will view questions about depression with four different response formats. Your task is to look at the questions and response formats so that you understand the questions in the second part of the study. The response formats are as follows: fill out a *Rating* scale, *Select* 5 descriptive words from a list of 30 words, write 5 descriptive *Words*, and write a *Text* of 20 to 1000 words. Second, you will be asked a series of questions about these response formats.

The questions in Phase 2 for respondents (a) and clinicians (b) are grouped according to how they are connected to the hypothesis.

Hypothesis 1: Precision

1 a. How much did the response format allow you to **elaborate**?

b. How much do you think the response format allowed the respondents to elaborate?

2 a. How **precisely** could you communicate your true feelings and symptoms in the following response formats:

b. How precisely do you think the respondents could communicate their true feelings and symptoms in the following response formats:

3 a. To what extent did the response format make you **think** through your problems related to depression?

b. To what extent do you think the response format made the respondent think through their problems related to depression?

4 a. To what extent did the response format evoke or **reinstate** emotions?

| b. To what extent do you think the response format evoked or reinstated emotions in the respondents? |
| --- |

5 a. How well could you communicate the **nature** of your depression, or lack of depression, in the following response formats?

b. How well do you think the respondents could communicate the nature of their depression, or lack of depression, in the following response formats?

6 a. To what extent did the response format make you think about your depression in a **different** way?

b. To what extent do you think the response format made the respondent think about their depression in a different way?

7 a. How **natural** was it to communicate your feelings using the following response formats?

b How natural do you think it was for the respondent to communicate their feelings using the following response formats?

8 a. How well could you **relate** to answering the question in the following response formats:

b. How well do you think the respondents could relate to answering the question in the following response formats:

Hypothesis 2: Easy

9 a. How **easy** was it to respond using the following response formats:

b. How easy do you think it was for the respondents to respond using the following response formats:

10 a. How **demanding** was it to respond in the following response formats:

b. How demanding do you think it was for the respondents to respond in the following response formats:

11 a. How **fast** could you respond in the following response formats:

b. How fast do you think the respondents could respond in the following response formats:

Hypothesis 3: Preference

12 a. Given you feel very depressed, to what extent would you **prefer** to use the following response format while communicating with a clinician?
