# Supplementary material for: Precise language responses versus easy rating scales—Comparing respondents’ views with clinicians’ belief of the respondent’s views
Source: PLoS One. 2023 Feb 15;18(2):e0267995. doi: 10.1371/journal.pone.0267995 (PMC9931093; doi:10.1371/journal.pone.0267995)
Supplement: Attachment — Submitted filename: reviewEY.docx [file pone.0267995.s002.docx]

I read this manuscript with a lot of interest. Being a researcher and clinician, I am always interested in methods that may improve assessment accuracy and validity. The authors of this study suggest that allowing respondents to express their experience using natural language reflect their experiences with more precision and elaboration. The authors also suggest that this method does not compromise diagnostic accuracy. They also conclude that when compared to clinicians, individuals who participated in the study preferred communicating their experiences in an open text format as opposed to closed format.

This paper opens a discussion for clinicians and researchers and raises important questions that may contribute to literature and advance knowledge. The application of their methodology to clinical samples is also novel and warrants further research and development.

However, I have several fundamental questions about the conclusions that authors draw from the results and a series of various issues that I am going to address.

The finding that respondents found the open-ended format responses to be a more precise depiction of their experience is hardly surprising. In clinical settings close ended rating scales are used as screening tools, provide an estimation for symptom severity, and can also be used to gauge progress. However, a diagnosis is never based on a screening tool alone. In general clients undergo a structured or semi structured interview conducted by a clinician who prompt them to describe their experience in their words. Only after careful elaboration a diagnosis can be provided.

Thus, the first fundamental question pertains to the clinical utility that open-ended format questions can offer given that 1. It is already accomplished though the interview part of the diagnostic process (MINI, or semi structured interview that is conducted when diagnosing the client) 2. Authors mention that this method does not seem to offer incremental validity (given the high correlation with validated existing scales). Did authors mean to imply that open ended questions may provide a richer data and a more useful information in *research settings* but may not necessarily be useful in *clinical settings?* Do authors suggest that open-ended format questions may be used in clinical settings as they may save on time and resources when no clinician is available to conduct the clinical interview?

My second fundamental question pertains to “precision and accuracy”, the perception that client’s account is precise and accurate may not always be relevant to the diagnostic criteria. For example, in some individuals with mental health conditions, perceptions of reality can be distorted, can include a psychotic process, can be irrelevant and overinclusive. Open ended questions may not be suitable for some individuals with OCD related disorders etc. Why do authors believe that perception of precision adds to incremental validity? Will this information be better assessed during clinical interview by a clinical psychologist or a diagnostician?

The third fundamental question pertains to the process of gathering information in vulnerable populations. For example, Jon Frederickson in his book on dynamic therapy techniques (p205) reminds clinicians that “Getting information without regulating the fragile patient’s anxiety would be harmful, not healing”. In the event where respondents were “reinstating or evoking” emotions, or “thought differently about their experiences” were they provided with interventions or strategies to manage distress, avoid re-traumatizing in case of PTSD or trauma related disorder, manage suicidality etc?

The fourth fundamental questions related to study population; a degree of cognitive impairment has been documented in individuals with MDD. Slower processing speed, memory issues, and other cognitive domains may be affected during depressive episodes. Is it possible that their accounts might not be factually precise?

Other issues I wanted to address briefly:

1. Title: I am not convinced that the title reflects study results. I don’t see how precise language responses challenge the rating scales. Close ended scales are used for a particular purpose and in *particular context.* They are not intended to capture the precise experiences that are highly individual for each respondent.
2. It is no doubt that short close ended questionnaires have limitations. Some are constrained by limited questions, some do not capture the full symptomatology (for example, PHQ has been shown to fail to identify depressed individuals with dysthymia). However, despite the limitations short screening measures have been shown to have high validity, can be normed, and using methods such as ROC can be used to establish meaningful cut-offs. Can authors discuss ways that NLP methodology using the open-ended questionnaires can used in a way that captures the severity of symptoms in a clinically meaningful way (cut-offs, severity, norms etc).
3. Short format close ended scales can also be advantageous as they can capturing most domains of symptomatology (somatic, cognitive, behavioural). For example, individuals with MDD may not fully be aware that lack of appetite, difficulty concentrating and decreased sex drive can be part of depression and thus not mention this in their description. Individual with MDD may also have difficulty retrieving information from memory and have slower mental processing speed. It is possible that some of these individuals will not be able to retrieve information that is pertinent. Short close ended scales such as BDI captures these aspects in case these will be missed. Can authors discuss how to address the issue of information that may be missed or underreported, but nonetheless be an important part of diagnosis.
4. It appears that this method will require an exclusion criteria ( i.e, low IQ, comorbid health conditions, psychotic processes, PTSD, cognitive impairment due to MDD or other conditions etc). Will that be a significant limitation?
5. Can authors comment on cultural factors that may affect the validity of their methodology ( more somatic presentations in some populations, non-disclosure etc)
6. In introduction the authors reference a study that was not published yet, to suggest that their method was validated by SADS, and is similar to results obtained through MINI or equivalent semi structured or structured interview. If SADS and MINI accomplish the same goal what advantage does NLP methodology offer above and beyond existing measures? Also, is it possible that some of the diagnoses were missed through PHQ and GAD is based on the fact that these are screeners and cannot be used as stand-alone diagnostic tools?
7. I have few comments regarding the choice of words: perhaps instead of “normal” controls, controls can be described as individual who did not meet the criteria for MDD or GAD. Other examples include the word “attitude” that I believe is meant to suggest preference, or perception, or biases (in methods, and discussion components).
8. While this method seems to be relatively new in clinical settings, can authors provide more references where it was evaluated in clinical samples?
