# Supplementary material for: Precise language responses versus easy rating scales—Comparing respondents’ views with clinicians’ belief of the respondent’s views
Source: PLoS One. 2023 Feb 15;18(2):e0267995. doi: 10.1371/journal.pone.0267995 (PMC9931093; doi:10.1371/journal.pone.0267995)
Supplement: Attachment — Submitted filename: Action letter PLOS ONE 2022-11-30_OKcomments.docx [file pone.0267995.s004.docx]

Dear Maki Sakamoto

Please find below our rebuttal letter for the manuscript PONE-D-22-11336R1

“Precise Language Responses versus Easy Rating Scales - Comparing Clinicians’ and Respondents’ Views”.

We have carefully addressed all constructive comments from the Reviewer and believe that the manuscript now is acceptable for publication in *PLOS ONE*.

Se our detailed comments below:

Reviewer #2: I want to thank the authors for the time and effort put into addressing the previous comments from myself and other reviewers. The additions and changes made have significantly improved the text, such as the inclusion of a section on Ethics as well as elaboration on discussion points. However, there are still a few issues that I would like to bring up that, I think, remain important and relevant to address.

Major comments:

I feel that there is still some key information lacking regarding the study sample that would help to situate the reader in their understanding of the study:
- Please provide more information (i.e., evidence from the literature) regarding the authors' interest in looking at the perceptions of clinicians regarding what their patients want. Although there is a huge body of literature in mental health about patient expectations, clinicians do not make clinical decisions solely on their patient's preferences (especially in a context such as assessment).

Our response: We have motivated the reason for why looking at response view by adding this in the abstract:

“…however, the respondents’ views as to what best communicates mental states are frequently ignored, which is important for making them comply with assessment.”

Further down in the introduction we have added:

“To study the respondents’ views of the assessment is important, as a working relation between patients and clinician over time is essential for motivating patients to reveal sensitive information.”

- Please elaborate further on the authors' decision to include individuals with GAD in the sample. I do not feel that being part of the population in previous studies is an acceptable reason for including these 21 participants.

Our response: We have removed the claim that the choice of looking at GAD is related to samples in previous studies, and replaced it with the argument that GAD and MDD has a large comorbidity:

”We chose to include participants with MDD or GAD as these are two major mental health disorders with a high comorbidity (i.e., rating scales of measuring them correlates highly).”

- Please elaborate on the recruitment of respondents (e.g., how many people met inclusion criteria? How were potential participants chosen? How many people dropped out? When did recruitment stop?)

Our response: We have now specified that 92 participants on Prolific met the inclusion criteria in the clinician group; and that of those 62 started the survey, 40 completed it, and 22 dropped out:

”There were 92 participants on Prolific that met the criteria for taking part in the clinician condition, where 62 of these participants started the study and 40 completed it (22 dropped out prior to completing the study.)”

In the respondents condition we have clarified that:

”We chose to include participants with MDD or GAD as these are two major mental health disorders with high comorbidity (i.e., rating scales of measuring them correlates highly). Participants that matched the pre-screened condition were invited to participate until the pre-defined number of participants was reached. The data collection lasted for approximately 17 hours on Prolific. Data of participants that did not complete the study was not saved.”

The inclusion of a paragraph on cultural factors is a really great addition to the paper, and would benefit from being expanded upon. Namely, the authors could mention the issues related to sexism and racism historically associated with machine learning (such as those discussed in The Alignment Problem by Brian Christian) and the consequences these might have for non-WASP individuals assessed using QCLA.

My only other major comment is that I feel the manuscript currently relies heavily on previous research conducted by this research group. I understand that there probably aren't many researchers conducting this kind of research (i.e., on QCLA); however, the manuscript often makes claims or refers do more general domains that would benefit from references outside the research group (such as asking general versus specific open-ended question).

We have added a paragraph that refers to more general domains and that discusses the point raised by the Reviewer:

“Another concern is that machine learning that is used by QCLA may be influenced by biases. For example, women are more likely to experience anxiety, leading to the concern that the machine learning may incorrectly use female language as evidence for anxiety. This effect has been labelled the Alignment Problem (Christian, 2022). Depending on the training data, this problem may influence various ethnical groups, and may be a disadvantage that may be particularly problematic for non-White Anglo-Saxon Protestants (WASP). “

Minor comments:

Please rewrite in the title and abstract to clarify that the study assessed clinicians' perceptions of what respondents preferred, rather than clinician preferences. This is well done in the remainder of the manuscript.

Our response:

The title has been changed to:

” Precise Language Responses versus Easy Rating Scales - Comparing Respondents’ Views with Clinicians’ belief of the Respondent’s Views”

Two sentences have been changed in the abstract:

"This was compared with the clinicians’ (*N* = 40) belief of the respondent’s view.”

” Respondents preferred the free text responses to a greater degree than rating scales compared to clinicians’ belief of the respondents’ views.”

Please clarify what are primary and secondary symptoms in the DSM-5; I am a clinician who regularly uses the DSM-5 and am not familiar with these terms.

Our response: We have removed the concept of ”primary and secondary” symptoms in DSM-5.

I am not sure that I understand why the following statement is included in description of clinician participants: "compare them with a respondent group that did not work professionally with mental health." My understanding of the sample is that there is no inclusion of professionals that have not worked in mental health.

Our response: This part of the sentence has been removed.

The following statements in the discussion is outside the scope of the current study and should be removed:
- "Together these findings open up for possibilities to use open-ended responses without sacrificing accuracy in measuring different constructs."
- the paragraph before the limitations section

Our response: This sentence and this paragraph have been removed.

Please clarify how the advances in NLP and QCLA would impact patients rating free text as higher than rating scales (on page 12 of the discussion).

Our response: We have changed the first sentence in this paragraph to:

” The findings that participants rate free text response to be more precise in communicating mental health compared to rating scales is consistent with recent findings showing that QCLA has a high validity in measuring mental health.”
